# A Jaw-Dropping Predicament

**Published:** 2012-11-27

**Authors:** Priti P. Patel, Ahmed M. S. Ibrahim, Jacob Zhang, John T. Nguyen, Samuel J. Lin, Bernard T. Lee

**Affiliations:** Beth Israel Deaconess Medical Center/Harvard Medical School, Boston, Mass.

**Figure:**
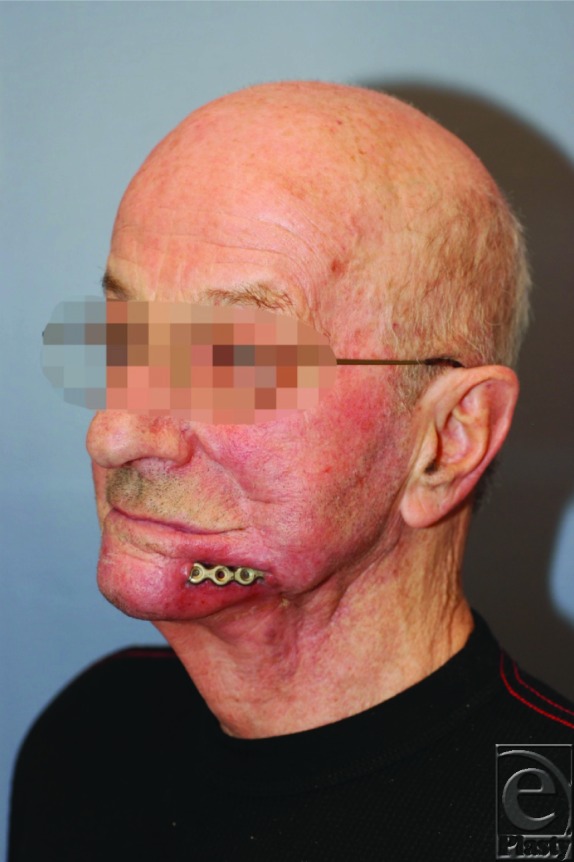


## DESCRIPTION

A 71-year-old man with history of asthma, non-insulin-dependent diabetes mellitus, and chronic obstructive pulmonary disease was treated for squamous cell carcinoma of the oral cavity. He underwent a partial mandibular resection with plate reconstruction followed by subsequent radiation therapy. A neck dissection was performed during the initial operative procedure. Eight months later, the patient was noted to have significant pain and development of intraoral fistulas. In addition, there was jaw swelling, difficulty opening the mouth fully (trismus), and bone destruction, which resulted in exposure of the mandibular reconstruction plates with evidence of underlying bony necrosis.

## QUESTIONS

1. **What is the most likely diagnosis for this patient?**
2. **What management options are available for treatment of this condition?**
3. **How does prior biphosphate therapy influence this condition?**
4. **How does hyperbaric oxygen (HBO) therapy affect the success rate of surgery?**

## DISCUSSION

Osteonecrosis is a nonfatal bone condition in which damage and/or necrosis occurs as a result of poor local blood supply. Osteonecrosis occurs in 1 in 100,000 people (0.001%), affecting 20,000 new patients a year typically between the ages of 20 and 50 years.^1^ It is often seen in the hip and shoulder, as well as the mandible. If untreated, affected bone may die and collapse. Common causes of osteonecrosis include diseases, such as sickle cell and diabetes, traumatic causes, and alcohol abuse. Often it is caused by radiation therapy, whereas it is called osteoradionecrosis (ORN).^2^

Prior bisphosphonate therapy has also been implicated as a cause of bone necrosis (bisphosphonate-associated osteonecrosis of the jaw) particularly at the lingual aspect of the posterior mandible.^3^^-^5 It is used in the treatment of several medical conditions, including bone metastasis associated with cancer, Paget's disease of bone and osteoporosis. However, its composition of pyrophosphate analogues reduces bone resorption and turnover resulting in the weakening of bone, especially when administered intravenously over a long period of time.

Osteoradionecrosis is caused by damage done by radiation to small arteries, depriving the bone of oxygen. It can be caused spontaneously when bone decays at a faster rate than it is created. It can also result when irradiated bone is subjected to trauma, most commonly tooth extraction (84%) and oncological surgery (12%). Symptoms include pain, swelling, inability to properly open the jaw (trismus), malocclusion, and exposed bone and pathologic fractures.^2^^,^^6^^,^^7^

Specifically, ORN of the mandible is characterized by 3 grades of severity. Grade I ORN is the most commonly observed, where the alveolar bone is exposed. This can be treated with HBO therapy, a process in which the patient is given 100% oxygen at hyperbaric pressures. Oxygen can diffuse into the bloodstream at a faster rate than at normal atmospheric pressure to restore vitality in the affected bone.^2^^,^^8^ However, its use has not yet been supported by a well-designed randomized clinical trial.^9^ Furthermore, to determine the safety and efficacy of using hyperbaric oxygen in the treatment of mandibular ORN, Annane et al^10^ reported that of 68 patients enrolled in their study, 6 showed improvement with HBO therapy while 12 improved on exposure to a placebo suggesting that patients with ORN do not benefit from HBO. However, it continues to be used readily because of anecdotal reports.

Grade II ORN is defined when the condition can no longer be treated effectively with HBO therapy. This usually requires the removal of dead bone (sequestrectomy) and/or the excavation of tissue to drain the infected bone (saucerization).^2^

Grade III ORN is characterized by the presence of a pathologic fracture. This is often accompanied by frank bony necrosis.^11^ Treatment includes resection, fixation of the mandible with reconstruction plates, and closure of the wound.^2^^,^^12^ Following resection of significant portions of the mandible, the bony defect is reconstructed most commonly with vascularized autologous tissue. Free fibula flaps are often used as a means of reconstruction and provide a healthy wound base to optimize healing.^13^ Additional reconstructive options include the use of a local pectoralis myocutaneous flap or a radial forearm fasciocutaneous flap. Depending on the extent of necrosis, radical resection may be performed followed by microvascular free flap anastomosis. In a 10-year retrospective cohort analysis, Chang et al^14^ reported a 37% complication rate following surgery—5 hardware related, 5 flap-specific, 3 infections, and 1 nonunion. Despite this, the authors maintain that a better quality of life can be attained for these patients through a multidisciplinary team approach with adequate debridement, resection, and reconstruction.
